# An association of vertebral breast cancer metastasis and multiple myeloma, revealed by a spinal cord compression

**DOI:** 10.11604/pamj.2014.19.168.5166

**Published:** 2014-10-17

**Authors:** Abdelhakim Kherfani, Khalil Amri, Mahjoub Hachem, Leila Abid, Mouna Bouaziz, Mondher Mestiri

**Affiliations:** 1 Department of Adults’ Orthopedics Surgery, MT Kassab, Institute of Orthopedics, Tunisia; 2 Department of Histology and Pathology, MT Kassab, Institute of Orthopedics, Tunisia; 3 Department of Medical Imagery, MT Kassab, Institute of Orthopedics, Tunisia

**Keywords:** Myeloma, breast cancer, spinal cord compression

## Abstract

Authors describe the case of a patient with breast cancer and multiple myeloma as the second metachronous disease responsible for spinal cord compression. Synchronous occurrence of bone marrow breast cancer disease and multiple myeloma has not been described in the literature, as in this case. By presenting this case, we point to possible association between both diseases and the possible factors involved in the development of second malignant disease.

## Introduction

Multiple myeloma (MM) is a malignant homeopathy due to a plasma-cell proliferative disorder representing 1% of all neoplasm, and 10% of malignant homeopathy. The vertebral involvement in multiple myeloma is very common and can cause spinal cord compression in 5% of cases [1, 2]. Chronic antigen stimulation from infection or other chronic disease has been associated with an increased incidence of MM. Breast cancer, mainly if lately diagnosed, essentially provides spinal metastasis [3] and can thereby be responsible for spinal cord compression. The combination of two malignant lesions in the same vertebra is exceptional; we report a case report of a rare combination of two malignant tumors of the same vertebra responsible for spinal cord compression.

## Patient and observation

**Figure 1:**
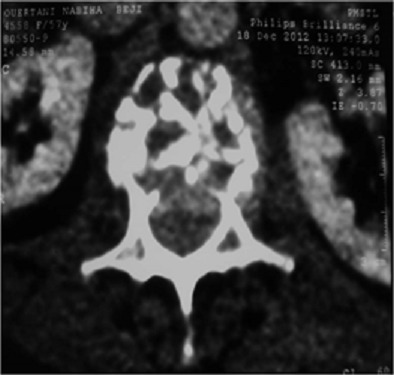
Axial computed tomography image demonstrating heterogeneities in the body of L2 with tumoral epiduritis

**Figure 2:**
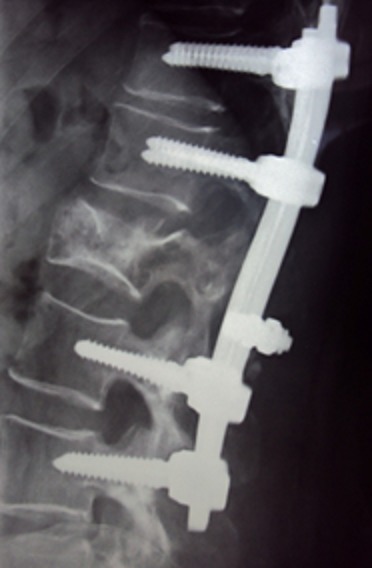
Post operative lateral view after synthesis from T12 to L4

A 57-year-old woman, with a medical history of hypothyroidism and hypertension, is followed since 2006 for infiltrating ductal carcinoma of the left breast, diagnosed at stage T2 N0 M0. The patient underwent a conservative surgery, with a regular 6-month follow-up. In November 2012, she consults for bilateral Lombosciatica evolving progressively since 4 months, with acute aggravation the last 48 hours, and installation of a motor deficit of the lower limbs touching the L3 to S1 roots. Thoraco-lumbar conventional radiography shows a vertebral compaction in L2 in butterfly wing, with rupture of cortical and decline in the posterior wall. MRI was not available; CT was made in emergency, confirming malignant settlement of L2 associated with tumoral epiduritis, compressing the dural sheath (Figure 1). L2 laminectomy, biopsy and posterior fixation from T12 to L4 were performed urgently (Figure 2). Complete recovery of the motor deficit followed the surgery within a couple of days.

**Figure 3:**
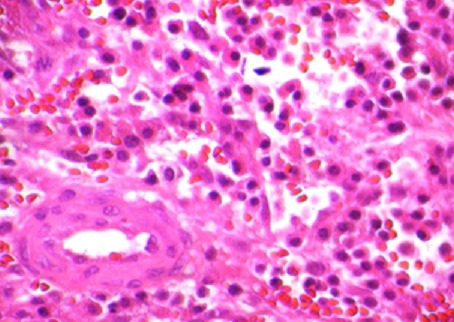
Histological features showing the two types of tumor cells

**Figure 4:**
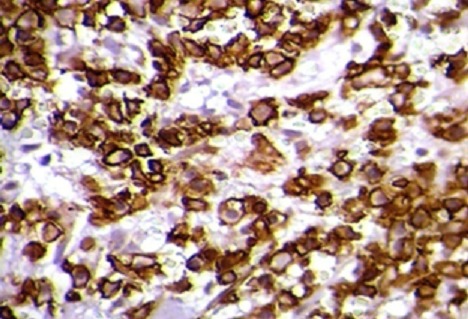
Immuno-histochemical results: CD138-positive plasmocytes (High power x 400)

Histopathological findings objectify the presence of tumoral cell, average-sized with hyper chromatic, irregular and atypical nucleus, corresponding to metastatic cells (Figure 3), associated with a lymphoplasmacytic inflammatory changes, CD138+, specific to multiple myeloma proliferation (Figure 4). The further investigations with complete laboratory tests (including SPEP and IEP) concluded to an IgG multiple myeloma at stage III A. The patient received additional adjuvant radiotherapy (a total of 10 sessions). At 12 months follow-up, the patient is doing well with a full motor function of the lower limbs (ASIA score: E) and a clinical, radiological and biological satisfactory state.

## Discussion

Incidence of multiple cancers varies in different patient populations and is related to geographical, race, age, environmental and genetic population factors. The coexistence of both neoplasias in a same tumor mass has been reported, but no cases of breast cancer metastases and MM of the same vertebra have been described. Among estrogen-dependent cancers, breast cancer is certainly the most widespread and the best studied. Worldwide, close to 1 million women per year are diagnosed with breast cancer.

Sebastien Maillard and other authors,[4] detected the presence of both estrogen receptor alpha and beta in MM cell lines, and showed that both a blockade of cell cycle and an increase of apoptosis were observed in MM cells lines with the use of anti-estrogens drugs, but the exact molecular mechanism of the enhancement of anti-estrogen activity following their incorporation into stealth colloidal formulations is still under current investigation. Shah I A [5] described a case of combination of neoplasm of the breast, lung and esophagus. Sakai A [6], reported a case of association of multiple myeloma and clear cell carcinoma of the kidney, and has advanced the hypothesis of a local secretion of growth factor especially IL-6 and its participation in the induction of multiple myeloma. Other interleukins, such as IL 10 and 11 have been implicated in the proliferation of lymphoid lineage [7]. Motzer [8] speculated genetic mutation that may cause coexistence of two populations of neoplastic cells.

## Conclusion

The Combination of multiple myeloma and breast cancer seems to be very rare. No cases of spinal cord compression due to a concomitant proliferation of metastatic breast tumor and malignant plasma-cell proliferation have been described. The study of this case should prompt us to seek common etiopathogenic factors between these two malignancies, and we encourage at least, practicing a dosage of growth factors in tissue from biopsy or even a genetic study research for a specific mutation.
